# Supplementary material for: qlifetable: An R package for constructing quarterly life tables
Source: PLoS One. 2025 Feb 21;20(2):e0315937. doi: 10.1371/journal.pone.0315937 (PMC11845031; doi:10.1371/journal.pone.0315937)
Supplement: S1 Annex — (DOCX) [file pone.0315937.s001.docx]

**S1 Annex: R code for adapting datasets in DEMOSPA0521 for use with qlifetable functions.**

The following code loads all datasets, corrects labels for age- and season-quarters, filters by sex and year, and selects appropriate columns to create datasets formatted similarly to those generated by the functions in Table 3, containing the relevant data for our example. This results in the creation of datasets t.p17, t.p18, t.b17, t.b18, t.d17, t.d18, t.e17, t.e18, t.i17, t.i18, d17, and d18.

R> # Loading of the datasets

R> t.pop <- read.csv("https://links.uv.es/bD1e56r", sep = ";", stringsAsFactors = TRUE)

R> t.births <- read.csv("https://links.uv.es/o4UU9bb", sep = ";", stringsAsFactors = TRUE)

R> t.deaths <- read.csv("https://links.uv.es/aqn2Pzb", sep = ";", stringsAsFactors = TRUE)

R> t.emi <- read.csv("https://links.uv.es/oekmAk1", sep = ";", stringsAsFactors = TRUE)

R> t.immi <- read.csv("https://links.uv.es/t0d9FBu", sep =";", stringsAsFactors = TRUE)

R> deaths <- read.csv("https://links.uv.es/rS16Fdf", sep = ";", stringsAsFactors = TRUE)

R> # Corrections of labels

R> t.pop$quarter_age <- as.numeric(t.pop$quarter_age)

R> t.births$quarter_age <- as.numeric(t.births$quarter_age)

R> t.deaths$quarter_age <- as.numeric(t.deaths$quarter_age)

R> t.emi$quarter_age <- as.numeric(t.emi$quarter_age)

R> t.immi$quarter_age <- as.numeric(t.immi$quarter_age)

R> deaths$quarter_age <- as.numeric(deaths$quarter_age)

R> t.pop$quarter_calendar <- as.numeric(factor(t.pop$quarter_calendar,

R> + levels = c("Winter", "Spring", "Summer", "Autumn")))

R> t.births$quarter_calendar <- as.numeric(factor(t.births$quarter_calendar,

R> + levels = c("Winter", "Spring", "Summer", "Autumn")))

R> t.deaths$quarter_calendar <- as.numeric(factor(t.deaths$quarter_calendar,

R> + levels = c("Winter", "Spring", "Summer", "Autumn")))

R> t.emi$quarter_calendar <- as.numeric(factor(t.emi$quarter_calendar,

R> + levels = c("Winter", "Spring", "Summer", "Autumn")))

R> t.immi$quarter_calendar <- as.numeric(factor(t.immi$quarter_calendar,

R> + levels = c("Winter", "Spring", "Summer", "Autumn")))

R> deaths$quarter_calendar <- as.numeric(factor(deaths$quarter_calendar,

R> + levels = c("Winter", "Spring", "Summer", "Autumn")))

R> # Datasets corresponding to year 2017

R> t.p17 <- t.pop[t.pop$gender == "Woman" & t.pop$valuation_year == 2017,

R> + c("age", "quarter_age", "quarter_calendar", "time_exposed")]

R> t.b17 <- t.births[t.births$gender == "Woman" & t.births$valuation_year == 2017,

R> + c("age", "quarter_age", "quarter_calendar", "time_exposed")]

R> t.d17 <- t.deaths[t.deaths$gender == "Woman" & t.deaths$valuation_year == 2017,

R> + c("age", "quarter_age", "quarter_calendar", "time_not_exposed")]

R> t.e17 <- t.emi[t.emi$gender == "Woman" & t.emi$valuation_year == 2017,

R> + c("age", "quarter_age", "quarter_calendar", "time_not_exposed")]

R> t.i17 <- t.immi[t.immi$gender == "Woman" & t.immi$valuation_year == 2017,

R> + c("age", "quarter_age", "quarter_calendar", "time_exposed")]

R> d17 <- deaths[deaths$gender == "Woman" & deaths$valuation_year == 2017,

R> + c("age", "quarter_age", "quarter_calendar", "number_events")]

R> # Datasets corresponding to year 2018

R> t.p18 <- t.pop[t.pop$gender == "Woman" & t.pop$valuation_year == 2018,

R> + c("age", "quarter_age", "quarter_calendar", "time_exposed")]

R> t.b18 <- t.births[t.births$gender == "Woman" & t.births$valuation_year == 2018,

R> + c("age", "quarter_age", "quarter_calendar", "time_exposed")]

R> t.d18 <- t.deaths[t.deaths$gender == "Woman" & t.deaths$valuation_year == 2018,

R> + c("age", "quarter_age", "quarter_calendar", "time_not_exposed")]

R> t.e18 <- t.emi[t.emi$gender == "Woman" & t.emi$valuation_year == 2018,

R> + c("age", "quarter_age", "quarter_calendar", "time_not_exposed")]

R> t.i18 <- t.immi[t.immi$gender == "Woman" & t.immi$valuation_year == 2018,

R> + c("age", "quarter_age", "quarter_calendar", "time_exposed")]

R> d18 <- deaths[deaths$gender == "Woman" & deaths$valuation_year == 2018,

R> + c("age", "quarter_age", "quarter_calendar", "number_events")]
